# Withdrawal of anticancer therapy in advanced disease: a systematic literature review

**DOI:** 10.1186/s12885-015-1862-0

**Published:** 2015-11-11

**Authors:** G. Clarke, S. Johnston, P. Corrie, I. Kuhn, S. Barclay

**Affiliations:** 1 Primary Care Unit, Department of Public Health and Primary Care, University of Cambridge, Cambridge, United Kingdom; 2 Carroll Lab Cambridge Research Institute, Cancer Research UK Cambridge Research Institute, Cambridge, United Kingdom; 3 Department of Oncology, University of Cambridge, Cambridge, United Kingdom; 4 Medical Library, University of Cambridge, Cambridge, United Kingdom

**Keywords:** Molecular targeted agents, Treatment decision-making, End of life, Palliative care

## Abstract

**Background:**

Current guidelines set out when to start anticancer treatments, but not when to stop as the end of life approaches. Conventional cytotoxic agents are administered intravenously and have major life-threatening toxicities. Newer drugs include molecular targeted agents (MTAs), in particular, small molecule kinase-inhibitors (KIs), which are administered orally. These have fewer life-threatening toxicities, and are increasingly used to palliate advanced cancer, generally offering additional months of survival benefit. MTAs are substantially more expensive, between £2-8 K per month, and perceived as easier to start than stop.

**Methods:**

A systematic review of decision-making concerning the withdrawal of anticancer drugs towards the end of life within clinical practice, with a particular focus on MTAs. Nine electronic databases searched. PRISMA guidelines followed.

**Results:**

Forty-two studies included. *How are decisions made?* Decision-making was shared and ongoing, including stopping, starting and trying different treatments. Oncologists often experienced ‘professional role dissonance’ between their self-perception as ‘treaters’, and talking about end of life care. *Why are decisions made?* Clinical factors: disease progression, worsening functional status, treatment side-effects. Non-clinical factors: physicians’ personal experience, values, emotions. Some patients continued treatment to maintain ‘hope’, often reflecting limited understanding of palliative goals. *When are decisions made?* Limited evidence reveals patients’ decisions based upon quality of life benefits. Clinicians found timing withdrawal particularly challenging. *Who makes the decisions?* Decisions were based within physician-patient interaction.

**Conclusions:**

Oncologists report that decisions around stopping chemotherapy treatment are challenging, with limited evidence-based guidance outside of clinical trial protocols. The increasing availability of oral MTAs is transforming the management of incurable cancer; blurring boundaries between active treatment and palliative care. No studies specifically addressing decision-making around stopping MTAs in clinical practice were identified. There is a need to develop an evidence base to support physicians and patients with decision-making around the withdrawal of these high cost treatments.

**Electronic supplementary material:**

The online version of this article (doi:10.1186/s12885-015-1862-0) contains supplementary material, which is available to authorized users.

## Background

Decision-making around starting and stopping treatment in advanced cancer is challenging for all concerned; patients, families and healthcare professionals alike. Appropriately timed cessation of treatment is an internationally recognised cancer treatment quality indicator [1–4], yet recent years have seen a trend towards continuing treatment until close to the end of life [5–7], at times within weeks of death [5, 7, 8]. In the UK, following a national audit of morbidity and mortality associated with chemotherapy, death within 30 days of receiving chemotherapy is now an accepted standard with an expectation that continuing chemotherapy in the last month of life is futile [9]. Recent research has demonstrated palliative cytotoxic chemotherapy at the end of life to be associated with a host of adverse outcomes including cardiopulmonary resuscitation, death in intensive care unit, place of death less likely to be at home or in preferred place and late hospice referral, as well as no survival benefit [10, 11].

Increased prescribing of cancer treatments over longer periods of time has been linked to availability of a plethora of new anti-cancer drugs, particularly the molecular targeted anticancer agents (MTAs) such as small molecule kinase inhibitors (KIs) [12, 13]. MTAs have been shown to improve outcomes in a variety of advanced malignancies, including chronic myeloid leukaemia, acute myeloid leukaemia, non-Hodgkin lymphomas, gastrointestinal stromal tumours (GIST), melanoma, renal and breast cancers [12, 14–16]. Notably, kinase-inhibitors (KIs) have improved survival in traditionally chemoresistant cancers, including advanced non-small cell lung cancer (NSCLC) [17–19], melanoma [20, 21] and renal cell cancer [22, 23].

Over recent years, MTAs have therefore transformed cancer management and their number is set to continue to increase over time [12, 14]. In addition to survival gains, oral MTAs allow for flexibility in care-planning, since patients administer their own treatment at home, avoiding hospital visits [24]. Although not without side-effects, MTA-induced toxicities are rarely life threatening with significantly lower rates of myelosuppression compared with conventional cytotoxic chemotherapy [14].

Eligibility to access MTAs is well defined by drug licensing indications, but stopping treatment, by contrast, is very different. RECIST (Response Evaluation Criteria In Solid Tumours) criteria are widely used within clinical trials for stopping treatment, based on proportional increase in tumour measurements during therapy [25]. Outside of trials, decision-making is far less tightly controlled and subjective endpoints influence decision making. Furthermore, recent evidence suggests that MTAs may improve survival despite RECIST evidence of measurable disease progression [26, 27]. Continued treatment with MTAs despite tumour growth is also being influenced by reports of rebound progression or “disease flare” on stopping therapy [20, 21, 28, 29]. There is also growing evidence of survival benefit from MTA re-challenge after a period off treatment in Non-Small Cell Lung Cancer (NSCLC) [28], and Gastrointestinal Stromal Tumour (GIST) [30] and renal cell cancer [31].

In most cases, the survival benefits of MTAs when treating patients with advanced solid tumours is measured in months rather than years [12, 14–16]. Costing between £2000 and £8000 per month, they impose considerable pressure on individuals and financially constrained healthcare systems. [32] There is therefore a strong health economic argument to ensure that guidance is available to support discontinuation of MTAs for reasons of futility. A systematic review of the literature was therefore undertaken, to identify current knowledge concerning decision-making in clinical practice to withdraw anticancer treatment in patients with advanced solid tumours.

### Aims

To examine decision-making concerning the withdrawal of anticancer treatments in patients with advanced solid tumours outside of clinical trials, with particular regard to MTAs:

1. How are these decisions made?
2. Why are these decisions made?
3. Who makes these decisions?
4. When are these decisions made?

## Methods

We undertook a systematic search of the international literature, utilising a thematic approach to analysis [33] and a narrative synthesis for presenting the findings [34].

### Search strategy and selection criteria

**Fig. 1:**
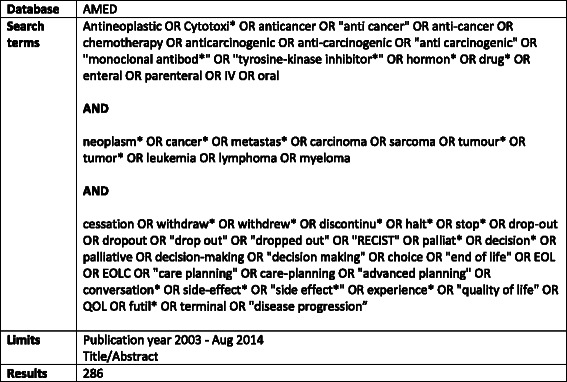
Cross-tabulation illustrating example search terms and strategy for an electronic database

Nine electronic databases (AMED (Allied and Complementary Medicine Database), ASSIA (Applied Social Sciences Index and Abstracts), BNI (British Nursing Index), CINAHL (Cumulative Index of Nursing and Allied Health Literature), Embase, MEDLINE, PsychINFO, SSCI (Social Sciences Citation Index), Cochrane Library) were searched for papers published between January 2003 and August 2013, using search strategies developed in conjunction with a professional librarian (IK) (Fig. 1). The grey literature was searched using the NHS evidence database, policy institute websites and charity and healthcare websites. Hand-searches of Lancet Oncology, British Journal of Cancer, Annals of Oncology and Journal of Clinical Oncology were undertaken, with reference and citation searches of all included papers. PRISMA guidelines were followed [35].

Inclusion criteria: studies of decision-making concerning cessation or continuation of oral or parenteral treatments for advanced malignant disease; qualitative or quantitative research methods; treatments being used in clinical practice; abstract or full paper available in English language. Studies investigating MTAs were particularly sought. Exclusion criteria: children under age 18, discussion or opinion pieces containing no new empirical data, steroids, bisphosphonates, treatment with radiation therapy only and clinical trial data.

### Data extraction, quality evaluation and analysis

Abstracts were screened by three authors (GC: social scientist, SJ: medical oncologist, SB: GP and palliative specialist) and full papers screened by GC and SJ who also independently assessed study quality using Gough’s Weight of Evidence framework [36]. Studies rated ‘high’ were given greater weight. A qualitative sensitivity analysis was performed [37]. We excluded studies rated as ‘low quality’ and assessed whether the findings were altered by this exclusion. Firstly, we examined whether any of the analytics themes were lost; and secondly we examined whether any of the ‘depth’ of the findings was lost. Data was entered into a review-specific data extraction form and then into NVivo9 for a narrative synthesis using a “thematic approach” [33].

### Ethics

This study did not require approval by an ethics committee as it is a systematic literature review of previously published work. PRISMA (Preferred Reporting Items for Systematic Reviews and Meta-Analyses) guidelines for a systematic review were followed [35].

## Results

**Fig. 2:**
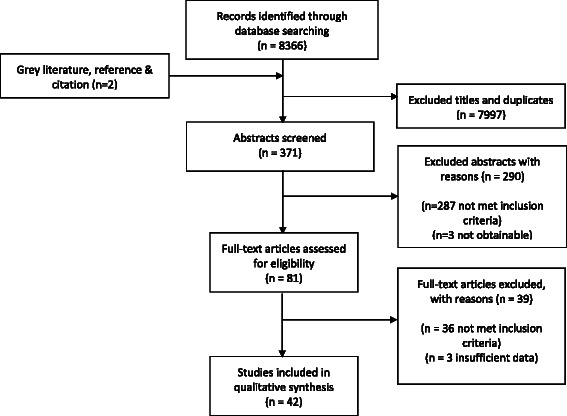
Flow diagram illustrating systematic search process

Searches yielded 8368 citations, 371 abstracts and 81 full papers were assessed. A total of 42 papers met the inclusion criteria and were included in the final analysis. Fig. 2 summarises this process.

Numbers of included papers are indicated in square brackets [ ]. Thirty-two papers (76 %) investigated decisions to stop treatment for a range of solid and haematological cancers. Ten (24 %) examined specific malignancies: lung [4], pancreas [2] and 1 each for ovarian, breast, lung and colorectal, colorectal and breast cancers. Most examined chemotherapy in general rather than specific drug regimens. Two papers discussed the use of MTAs in the final months of life [38, 39], and a further three briefly commented on MTAs [40–42]: none described decision making processes concerning stopping MTAs close to the end of life. Papers came from 14 different countries, some studies comprised evidence from multiple countries: USA [15], Netherlands [5], UK [4], Japan [3], 2 each from New Zealand, Canada, Italy and Sweden and 1 each from Germany, Australia, Taiwan, Belgium, Korea, UK with USA, and Australia with Israel. Twenty-four of these studies contained primarily qualitative data, and 18 contained primarily quantitative data. Additional file 1 [see Additional file 2] summarises the main characteristics of included studies. The sensitivity analysis revealed that removing studies rated as low quality did not alter the overall findings; no themes or topics were lost, and only a small amount of the ‘depth’ or ‘thickness’ of the findings was lost.

### (1) How are decisions made?

The process of treatment withdrawal was described as a shared progression of many different conversations rather than a one-off decision [11, 38, 40, 42–50]. It involved a trajectory of repeated reassessments and trying different treatment options [13, 38, 40, 42–50], including treatment breaks before starting subsequent new regimens [4, 38, 40, 42, 50]. MTAs contribute to this process by providing more options before cessation of all treatment [2, 38, 39]. Decisions about withdrawing treatment were described as an integral part of patient care [45], strongly influenced by patients’ views and wishes [51]. Collusion in continuing inappropriate treatment may occur unless both oncologist and patient are ready to discuss stopping treatment and both acknowledge that the end of life is near [38].

Oncologists commonly report finding discussions about stopping treatment difficult [6, 38, 42, 44, 46, 50, 52] and emotionally challenging [3, 38, 49, 52].

“*What I like least* [about my job] *is giving people bad news. It’s just terrible. It’s just horrible. It’s so sad…It’s just horribly sad*.” (Oncologist) [38]

Clinicians reported that discussions around stopping treatment are commonplace and challenging, with a limited evidence base outside of clinical trial protocols available for guidance [38]. The ‘grey area’ in which patients want another line of chemotherapy and their condition could permit it, but on the balance benefits and burdens it would be inadvisable, is particularly challenging [52]. The process of withdrawing treatment involves switching role from that of an “advocate” for curative or disease modifying treatments to a supportive “counselling” role for palliative, end of life care [49]. This role switch may create “role dissonance” for oncologists who often perceive themselves as “treaters” who “do something” [38]. Clinicians may become “stuck” on an “institutional script for treatment” [46] which prevents them from discussing alternative supportive interventions, “watchful waiting” [53], or discussions concerning death and dying [46, 54].

Stopping treatment may be raised by clinicians as a new direction of treatment in which the patient’s emotions are directly addressed [42], or by presenting it as a “biological fact” that leaves little room for discussion:

“*And so we started you off with the standard treatment. . . .We didn’t quite see what we wanted with that, we moved on to something else. One thing I just want to make sure you realize is that we don’t save our best weapons for the end. We use them up front. So the chances of you responding to other agents, are even less than the chances were with the two other regimens you’ve already gotten*”. (Oncologist talking to patient) [42]

Some oncologists describe a “contemplation stage” during which evidence is gathered concerning disease progression and treatment options and consideration given on how to present this to the patient [38, 49, 52].

Few studies addressed the involvement of patients and their supporters in treatment cessation decisions. Oncologists expressed the view that patients need to be beginning to accept their terminal status before they can be ready to consider stopping treatment [3, 44, 47, 51], for some this acceptance may not happen until very close to death [44]. Patients with greater experience of treatment may be more confident about negotiating treatment options and being involved in decisions [48]. Those with strong belief in the effectiveness of the treatment may have it continued longer [55]. Some patients withdraw from the decision-making process as their disease progresses and a surrogate decision-maker takes over [51, 53]. A perception that doctors do not wish to involve family members in discussions may cause considerable distress [56].

### (2) Why are decisions made?

Decisions to stop treatment are complex and multifaceted, with four major factors identified from the literature.

(a) *Disease and clinical factors:* Key factors in decisions to stop treatment included worsening patient condition or functional status [10, 38, 43, 44, 49, 52, 55, 57–60] disease progression or advanced stage disease [9, 38, 40, 43, 44, 47, 49, 52, 57, 60] and treatment side effects [7, 38, 49, 52, 55, 59–61]. Patients who received their diagnosis when only in advanced disease, and patients who had not received treatment earlier in the course of illness for other reasons, often had their therapy extended much longer than patients who had received earlier treatments [5, 38, 62–65].

The type of cancer influenced treatment decisions [7, 38, 43, 49, 60, 62, 64, 66]. For example, patients with haematological malignancies [4, 38, 43, 62, 63], and advanced lung cancers [2, 38, 64], were more likely to have their treatment continued. Chemo-responsiveness of tumours was reported to be a key factor in decisions to continue treatment [2, 43, 66], although two population-based studies found no such connection [41, 67]. Six population studies found treatment was more likely to be withdrawn or withheld in older people [57, 58, 62, 63, 67, 68], although one study found age was not a predictor of discontinuing palliative treatment [60].

(b) *Clinician-dependent factors:* A range of non-clinical factors influenced decision-making in the face of cancer progression. Clinicians’ personal approaches, heavily shaped by their personal perspectives and ethics [6, 38, 43, 49, 52, 69, 70] were significant predictors of whether chemotherapy was continued as disease progressed [2, 57, 66] Doctors’ views of patients’ personalities and circumstances also influenced the decision [4, 38, 43, 49, 70].

“*I think instinctively you feel that this is a young patient with a young family you need to make even more effort to try and help them live for a bit longer”.* (Oncologist) [49]

Some doctors reported they would continue treatment within two weeks of death for even a small chance of possible extension of life [39]. Treatment was also more likely to be continued by younger and less experienced doctors [2, 38, 43], and in the face of uncertainty of clinical benefit [38, 39].

“*We are poor predictors of prognosis even in these near death time frame and we therefore err on the side of more treatment*.” (Oncologist) [39]

Treatment was often continued to avoid “taking away hope” [4, 38, 49, 52, 71] and when strong relationships have developed with patients [3, 43, 44, 72].

(c) *Patient-dependent factors:* Hope for the future was an important driver for patients’ decision-making [3, 44, 47, 48], although this hope at times reflected poor understanding of the palliative rather than curative aims of treatment [4, 48, 72–75]. When making decisions, patients strive to balance hope and improvements in quality of life, with side-effects and the burdens of treatment [5, 51, 55, 61, 63, 68]. Older patients are more likely to discontinue treatment when approaching the end of life [8, 57, 58, 62–64, 66, 67], although one study found no such association [60].

(d) *Environmental factors:* Hospital setting was a key influence on whether or not treatment was continued [6, 38, 39, 57, 60, 62, 76, 77]. Treatment was more likely to be continued in teaching hospitals [62], and those receiving private treatment in physicians’ offices, rather than in general hospital outpatient clinics [77]. Access to and information about palliative care services was associated with stopping treatment [3, 39, 57, 60], as was having a supportive care plan in place [54], although hospice referral was not always associated with treatment discontinuation [54].

Treatment costs also influence these decisions, particularly in health systems where patients have to meet drug costs personally [3, 38, 39, 77]. The availability of new MTAs, although expensive, influence decisions to continue treatment as they provide additional options for continuation of treatment [3, 38, 39, 43].

### (3) When are decisions made?

The literature concerning the timing of decisions to withdraw treatment was very limited. Clinicians expressed great difficulty over judging the appropriate time to stop [5, 38, 43, 44, 48, 49], particularly when a patient’s condition could justify, but the prognosis was unclear and the benefit of treatment was uncertain [52]. Patients also expressed uncertainty about the timing of treatment withdrawal. In one study, all participants unanimously emphasised the difficulty of anticipating the right time to make the decision to stop all treatment [48].

### (4) Who makes the decision?

The majority of studies reporting upon ‘who makes the decision’, found decision-making was an on-going process based in the interaction between the physician and patient, and sometimes close relatives [8, 38, 40, 43, 45, 48, 49, 52, 73]. However there was mixed evidence upon who in reality was involved in decision-making and who made the final decision. One study reported fewer patients to have been involved in the decision-making process than wanted to be, and that only half of competent patients were involved [78]. In contrast, another study reported family members to believe that the patients’ preferences had been followed in 78 % of cases [79]. Nurses often have an important supportive role [4, 43, 52, 57, 61], as may the wider multidisciplinary team [49].

A smaller number of studies found decisions were driven by one particular party: four studies found decision-making was patient driven, two of these studies were based in the USA, one was based in The Netherlands and the other in Israel and Australia [39, 44, 51, 52]; in contrast three studies found physicians had the strongest influence, one on these studies was based in the USA, one in New Zealand and one in the Netherlands [43, 52, 71]. The number of studies and the limited high quality evidence on this topic means that no effect by country or continent could be found.

Doctors exerted influence by choosing which options to present to patients [43], and the way in which those options were presented [52]. Some patients preferred not to be involved in the decision-making at all [48], while for others feeling in control was important, even if it was only control of day-to-day decisions [44, 48, 80].

“*I want to be able to have control or say about my illness, whether I think I should take chemo or not. The doctors tried to talk me out of it*[her decision to stop treatment]*, and it’s just like, it’s MY body. I feel it’s not going to do anything for me. It’s making me sicker so why do it? I felt that it was important for me to have control over that*.” (Patient declining chemotherapy in advanced disease) [80].

## Discussion

Deciding that “enough is enough”, that cancer treatment should be stopped in the face of approaching end of life, is one of the most challenging decisions that patients, their families and oncologists have to make. Based on limited published evidence primarily derived from the USA, these decisions appeared to be made over a period of time, in the context of an ongoing and trusting relationship between oncologist and patient, and were largely clinician-guided. Clinical factors such as disease response and progression dominated treatment decision-making, with physicians having to weigh up the potential “costs” of the treatment to the patient such as side-effects and toxicities, with potential “benefits” such extended life-span or giving the patient hope for the future.

The decision to continue or stop cancer chemotherapy has costs associated, both to the patient and the wider health economy. While MTAs have fewer life threatening toxicities than conventional cytotoxic chemotherapy, common toxicities including rash, diarrhoea and interstitial pneumonia generate hospital admissions [81, 82]. Multi-targeted Vascular Endothelial Growth Factor Receptor Tyrosine-Kinase Inhibitors (VEGFR TKIs) are associated with haemorrhage, hypertension, adrenal dysfunction, hypothyroidism and acute coronary syndrome [83]. Some BRAF (B-Raf proto-oncogene, serine/threonine kinase) targeted inhibitors are associated with chronic photosensitivity and risk of skin squamous cell cancers [84]. Many MTAs cause fatigue [14], which may significantly impair quality of life.

However, subtle factors such as clinicians’ attitudes and personalities, doctor-patient relationships and relatives’ and patients’ views strongly influenced decisions. A concern to foster ‘hope for the future’ was a significant factor for all parties, but at times, this hope was unrealistic, reflected poor understanding of palliative treatments, or arose from physician collusion. These findings show that the nature of the patient-physician relationship is a key part of decision-making concerning the withdrawal of anticancer drugs towards the end of life, and add growing support to the body of knowledge on the importance on ‘trust’ and ‘rapport’ in shared healthcare decision-making [85, 86].

To our knowledge, this is the first systematic review of decision-making concerning withdrawing anticancer treatments in advanced disease within clinical practice. We used a rigorous and inclusive search strategy to comprehensively search the international literature, including nine electronic databases and grey literature. A broad range of study populations and methodologies were included, incorporating evidence from both qualitative and quantitative studies. Several factors indicate the robustness of the findings: the themes identified were repeated across a large proportion of the studies, despite the heterogeneous contexts and populations; the themes did not vary substantially across different countries; and the sensitivity analysis revealed that removing studies rated as ‘low’ did not alter the findings of the review.

This review does have several limitations. There is limited high quality evidence: using Gough’s weight of evidence framework [36] only nine studies were rated as high quality, 15 as medium and 18 as low quality. Many of the low rated studies had a small sample size or limited evidence relevant to the review questions. Higher quality studies were given greater weighting in the thematic analysis, although sensitivity analysis revealed that removing studies rated as ‘low’ did not alter the findings of the review. The heterogeneous nature of included studies made comparisons difficult; we selected a thematic analysis and narrative synthesis methodology to account for this. Only studies published in English language were included: although the included studies had a wide international range, 38 % (16/42) of included papers were from Europe, 36 % (15/42) from the United States and 12 % (5/42) from Asia. Clinical practice may be particularly different in the US where patients may exert pressure to continue treatment and clinicians have personal financial incentives to prolong treatment.

## Conclusions

The advent of molecular targeted agents (MTAs) has brought new benefits as well as challenges to modern cancer therapy, potentially blurring the distinction between active and palliative interventions. Traditionally, disease progression is an indication to stop treatment, but outside of trials, stopping treatment is far more challenging. Furthermore, there is evidence to show that patients receiving oral MTAs continue treatment until closer to death than those receiving intravenous anticancer therapies [38, 39, 41, 69]. While it is generally considered that continuing treatment in the last 30 days of life must be futile from a biomedical perspective, there may be benefit in continuing MTA treatment beyond disease progression [26], particularly given the potential for “tumour flare” on stopping these therapies [26]. It has been suggested that for modern targeted therapies, the definition of disease progression may itself need redefining [16, 27]. It remains unclear how best to integrate MTAs into modern treatment algorithms [16, 27].

Wider subjective factors clearly influence treatment decision-making and the relative balance of harms and benefits associated with patients, carers and doctors choosing to continue anticancer drugs beyond disease progression has not yet been determined. While MTAs offer real hope for patients with advanced cancers, their considerable financial costs are raising major health economic and ethical concerns in resource-constrained health services across the world. With rising cancer incidence and increasing numbers of patients on MTA treatment across the world, it is important for clinical practice, as well as society as a whole, to have realistic expectations of these new agents. We found no studies which specifically addressed decisions concerning when to stop treatment with MTAs. While the recent development of these agents may in part be responsible for the lack of research, there is clearly a pressing need to develop an evidence-base to allow physicians to weigh up the potential ‘costs’ and ‘benefits' of these treatments and inform optimal decision-making associated with stopping MTAs.

## Additional file

Additional file 1: **Cross-tabulation illustrating evidence summary of included papers (*****N***** = 42).** (DOCX 48.2 kb)
Additional file 2: **PRISMA 2009 Checklist.** (PDF 91 kb)

## Abbreviations

MTA: molecular targeted agent
KI: kinase inhibitor
RECIST: response evaluation criteria in solid tumours
NSCLC: non-small cell lung cancer
GIST: gastrointestinal stromal tumour
VEGFR TKI: vascular endothelial growth factor receptor tyrosine-kinase inhibitors
BRAF: B-Raf proto-oncogene, serine/threonine kinase
PRISMA: preferred reporting items for systematic reviews and meta-analyses
AMED: allied and complementary medicine database
ASSIA: applied social sciences index and abstracts
BNI: British nursing index
CINAHL: cumulative index of nursing and allied health literature
SSCI: social sciences citation index
