# Supplementary material for: Withdrawal of anticancer therapy in advanced disease: a systematic literature review
Source: BMC Cancer. 2015 Nov 11;15:892. doi: 10.1186/s12885-015-1862-0 (PMC4641339; doi:10.1186/s12885-015-1862-0)
Supplement: Additional file 1: — Cross-tabulation illustrating evidence summary of included papers (N = 42). (DOCX 48.2 kb) [file 12885_2015_1862_MOESM1_ESM.docx]

**Table 1. Cross-tabulation illustrating evidence summary of included papers (N=42)**

| **No.** | **Reference** | **Country** | **Anticancer treatment** | **Cancer type** | **Participants** | **Method** | **Summary of findings** | **Weight of evidence** |
| --- | --- | --- | --- | --- | --- | --- | --- | --- |
| **1** | Bluhm M, V. Factors influencing oncologists' use of chemotherapy in patients at the end of life: A qualitative study. *Dissertation Abstracts International: Section B: The Sciences and Engineering* 2012; 72(8-B). | USA | Palliative chemotherapy/ Chemotherapy at EOL | All | Oncologists  (N=17) | Qualitative interview | 1) Patient has to have acceptance of terminal nature of disease to stop treatment  2) Oncologists have to feel ready to have discussion. Large emotional burden  3) Continuing to treat palliates both patient and oncologist  3) Transactional relationship between patient-physician is key  4) Stage of disease, pace of disease and previous treatment history are important  5) Environmental factors such as new drugs, financial incentives and time pressure also play a role | HHH - **H** |
| **2** | Buiting. Understanding provision of chemotherapy to patients with end stage cancer: qualitative interview study. *BMJ* 2011; 342:1933-1941. | Netherlands | Palliative chemotherapy/ Chemotherapy at EOL | All | Physicians (N=14) and Nurses (N=13) | Qualitative interview | 1) Physicians and nurses aim to inform patients’ about poor prognosis & treatment options  2) Physicians & nurses carefully consider the effects weighed against QOL  3) Physicians preserve patient wellbeing by offering further chemotherapy. Nurses had more doubt  4) Physicians may “try out one dose” if patient wants it in uncertain circumstances  5) Discussing dying at the same time as chemotherapy was considered contradictory | HHH – **H** |
| **3** | de Kort SJ, Pols J, Richel DJ, Koedoot N, Willems DL. Understanding Palliative Cancer Chemotherapy: About Shared Decisions and Shared Trajectories. *Health Care Analysis* 2010; 18(2):164-174. | Netherlands | Palliative chemotherapy/ Chemotherapy at EOL | All | Patients  (N=13) | Detailed longitudinal case studies: Includes:  Observations and qualitative interview | 1) Numerous treatment options continually tailored in patient-physician interaction (not just ‘one decision’)  2) Options could change e.g. move from taking a break in treatment to stopping  3) Treatment options kept open – not final | HHH - **H** |
| **4** | Behl D, Jatoi A. What do oncologists say about chemotherapy at the very end of life? Results from a semiqualitative survey. *Journal of Palliative Medicine* 2010; 13(7):831-835. | USA | Palliative chemotherapy/ Chemotherapy at EOL | All | Oncologists (N=61) | Semi-structured survey | 1) Decisions for chemo at EOL are strongly patient-driven  2) Newer agents are driving the decision to continue with cancer treatment  3) Financial incentives on the part of the medical community explain these high rates  4) Healthcare reform is necessary;  5) Even a small chance of patient benefit justifies this practice  6) Practice is detrimental to patients because it precludes the initiation of hospice services  7) Others may be prescribing in this manner, but ‘‘not us’’  8) Issues are complicated, revolve around society values, and the oncologist alone cannot be responsible | MHH - **H** |
| **5** | Koedoot CG, Oort FJ, De RJ, Bakker PJ, De A, De JC. The content and amount of information given by medical oncologists when telling patients with advanced cancer what their treatment options are palliative chemotherapy and watchful-waiting. *European Journal of Cancer* 2004; 40(2):225-235. | Netherlands | Palliative chemotherapy/ Chemotherapy at EOL | All | Patients (N=95) | Qualitative interview and observations | 1) Physicians tell their patients little about watchful-waiting discussions  2) Physicians give older people and married people more information about treatment  3) Physicians working in academic hospitals gave more information  4) Amount of information given did not affect decision outcome | HMH - **H** |
| **6** | McCullough L, McKinlay E, Barthow C, Moss C, Wise D. A model of treatment decision making when patients have advanced cancer: how do cancer treatment doctors and nurses contribute to the process? *European Journal of Cancer Care* 2010; 19(4):482-491. | New Zealand | Palliative anticancer treatment/ at EOL | All | Doctors  (n= 8) and nurses (n=13) | Qualitative interview | 1) Doctors choose which options to offer, then patient makes decision, nurses have supportive role  2) Decision-making is cyclical process – in which treatment outcomes are evaluated and options changed  3) Younger doctors and those with least experience are most likely to continue to treat | MHH - **H** |
| **7** | Meeker MA. Responsive Care Management: Family Decision Makers in Advanced Cancer. *Journal of Clinical Ethics* 2011; 22(2):107-122. | USA | Palliative anticancer treatment/ at EOL | All | Family surrogates/care-givers (N=40) | Qualitative interview | 1) Decisions embedded in other care-giving  2) Decision style change during course of illness from the care-giver in a supportive role to taking over decisions  3) Split between participants who wanted to make own decision and those who wanted clinician guidance | HMH - **H** |
| **8** | Schildman J, Ritter P, Salloch S, Uhl W, and Vollman J. 'One also needs a bit of trust in the doctor ... ‘ a qualitative interview study with pancreatic cancer patients about their perceptions and views on information and treatment decision-making. *Ann Oncol* epub. 2013. | Germany | Palliative chemotherapy/ Chemotherapy at EOL | Pancreatic | Patients (N=12) | Qualitative interview | 1) Hope is an important driver in decision to continue  2) No difference in ‘hope’ between early and late stage patients – ‘illusion’ around cancer  3) Difficult to anticipate timing of stopping  4) As patient becomes more experienced with treatments they take larger role in decision-making | MHH - **H** |
| **9** | Weeks C, Catalano J, Cronin A, Finkelman D, Mack W, Keating L et al. Patients' expectations about effects of chemotherapy for advanced cancer. *The New England Journal of Medicine* 2012; 367(17):1616-1625. | USA | Palliative chemotherapy/ Chemotherapy at EOL | Lung and/or colorectal | Patients (N=1193) | Prospective cohort study: Interviewer guided computer-assisted interview | 1) Inaccurate beliefs. Overall, 69% of patients with lung cancer and 81% of those with colorectal cancer did understand that chemotherapy was not at all likely to cure their cancer  2) Risk of reporting inaccurate beliefs higher among patients with colorectal cancer, as compared those with lung cancer  3) Educational level, functional status, and the patient’s role in decision making were not associated with such inaccurate beliefs about chemotherapy | HMH - **H** |
| **10** | Back L, Michaelsen K, Alexander S, Hopley E, Edwards K, Arnold M. How oncology fellows discuss transitions in goals of care: A snapshot of approaches used prior to training. *Journal of Palliative Medicine* 2010; 13(4):395-400. | USA | Palliative chemotherapy/ Chemotherapy at EOL | All | Palliative care physicians  (N=20) | Observation of physicians discussing patient vignette | 1) Some physicians discussed the limitations of anticancer treatment as a scientific fact using biomedical logic  2) Some physicians put patients’ reactions to stopping treatment as central to discussion  3) Some physicians offered new direction for medical care now that anticancer had been exhausted or offered emotional solutions | HMM - **M** |
| **11** | Maida V, Peck J, Ennis M, Brar N, Maida AR. Preferences for active and aggressive intervention among patients with advanced cancer. *BMC Cancer* 2010; 10. | Canada | Palliative chemotherapy/ Chemotherapy at EOL | All | Patients and substitute decision-makers (n=380) | Questionnaire | 1) 61.9% of decided patients expressed the desire to withhold further chemotherapy if offered  2) 38.1% wanted chemotherapy at the end of life  3) Those who wanted it were more likely to be: younger, non-Caucasian, have a higher performance status, to have higher CCI, and to have a SDM involved in the decision | HMM - **M** |
| **12** | Pardon K, Deschepper R, Vander Stichele R, Bernheim JL, Mortier F, Schallier D et al. Preferred and Actual Involvement of Advanced Lung Cancer Patients and Their Families in End-of-Life Decision Making: A Multicenter Study in 13 Hospitals in Flanders, Belgium. *Journal of Pain and Symptom Management* 2012; 43(3):515-526. | Belgium | Palliative anticancer treatment/ at EOL | Non-small cell lung | Patients (N=85) | Questionnaire | 1) Only half of competent patients involved  2) Fewer involved than wanted to be  3) Palliative goal more likely to be involved that continuing with life lengthening palliative treatment | MHM - **M** |
| **13** | Schildman J. "Well, I think there is great variation...": a qualitative study of oncologists' experiences and views regarding medical criteria and other factors relevant to treatment decisions in advanced cancer. *Oncologist* 2013; 18(1). | UK | Palliative chemotherapy/ Chemotherapy at EOL | All | Oncologists (N=12) | Qualitative interview | 1) Evidence is scarce about time scales – creates difficult decision-making  2) Main clinical factors: “ “diagnosis,” the “stage of disease,” “patients’ health status,” and “available treatment” as the usual  “medical and clinical decision criteria”  3) Non-clinical factors: Physicians own personal judgment/values and Physicians’ perceptions of patients’ ages and circumstances | MMH - **M** |
| **14** | Volker DL and Wu H-L. Cancer Patients' Preferences for Control at the End of Life. Qualitative Health Research 21(12), 1618-1631. 2011. | USA | Palliative anticancer treatment/ at EOL | All | Patients (N=20) | Qualitative interview | 1) Patients with experience of loved ones in similar situations are more likely to want to stop  2) Patients worry about being a burden  3) Patients want control over decisions | MMH - **M** |
| **15** | Rose JH et al. ‘Perspectives, preferences, care practices, and outcomes among older and middle-aged patients with late-stage cancer’,  *Clin Oncol*. 2004 Dec 15;22(24):4907-17. | USA | Palliative anticancer treatment/ at EOL | All | Older (n=696) and Middle-Aged Patients (n=720) With  Late-Stage Cancer | Questionnaire | 1) Discussion of aggressiveness of care linked to doctor’s perception of survival in middle age and older age  2) Readmission and early death linked to doctor’s talking about limiting treatment in older and middle  3) Continuing treatment linked to patient’s own perception of prognosis not doctor’s for older and middle  4) Older patients who wanted pain relief only discussed fewer topics | HMM - **M** |
| **16** | Chouliara Z, Miller M, Stott D. Older people with cancer: perceptions and feelings about information, decision-making and treatment: a pilot study. *European Journal of Oncology Nursing* 2004; 8(3):257. | UK | Palliative anticancer treatment/ at EOL | All | Older people with cancer (N=6) | Qualitative interview | 1) Older people want to continue with cancer treatment as long as they had “average quality of life” – defined by: enjoying life, not suffering severe pain, cancer not a big disruption to normal everyday life, can occasionally put cancer-related worries aside  2) Older people with cancer were also capable of describing an organised decision-making process they use to evaluate different factors (e.g. side effects) and arrive at decisions. | MMM - **M** |
| **17** | Ohlen J, Elofsson LC, Hyden LC, Friberg F. Exploration of communicative patterns of consultations in palliative cancer care. *European Journal of Oncology Nursing* 2008; 12(1):44-52 | Sweden | Palliative anticancer treatment/ at EOL | All | Physicians, patients and relatives (N=16) | Observation | 1) Doctors led conversations - Physicians controlled conversation and stuck to a script dominated by institutional framing – mostly unchallenged. Patients initiated talk about the future  2) These agreed upon agendas may prevent physicians from discussing sensitive issues that patients wish to bring up | MMM - **M** |
| **18** | Andreis F, Rizzi A, Rota L, Meriggi F, Mazzocchi M, Zaniboni A. Chemotherapy use at the end of life. A retrospective single centre experience analysis. *Tumori* 2011; 97(1):30-34. | Italy | Palliative chemotherapy/ Chemotherapy at EOL | All | Patients with metastatic or advanced solid tumors  (N=102) | Medical records analysis | 1) Younger age not a predictor of continuing chemotherapy near the end of life  2) Chemotherapy more likely to be stopped if the patient lived in an area with access to palliative care services | MLM - **M** |
| **19** | Andrew J, Whyte F. The experiences of district nurses caring for people receiving palliative chemotherapy. *International Journal of Palliative Nursing* 2004; 10(3):110. | UK | Palliative chemotherapy/ Chemotherapy at EOL | All | District nurses  (N=10) | Qualitative interview | 1) Nurses provided information and support in patients decision-making  2) When treatment side-effects become burdensome and patient may wish stop, the established relationship with the DN provided reassurance that patient will be supported so patient can continue treatment  3) DNs have ambivalent attitude towards palliative chemo – privately they questioned the reasons behind burdensome treatment | MLM - **M** |
| **20** | Barthow C, Moss C, McKinlay E, McCullough L, Wise D. To be involved or not: Factors that influence nurses' involvement in providing treatment decisional support in advanced cancer. *European Journal of Oncology Nursing* 2009; 13(1):22-28. | New Zealand | Palliative anticancer treatment/ at EOL | All | Nurses (N=13) | Qualitative interview | 1) Some nurses actively involved in decision-making actively seeking out opportunities to be involved in decision-support – some no or minimal involvement  2) Older experienced nurses more likely to be involved in decision-support | MLM - **M** |
| **21** | Kacen L, Madjar I, Denham J. Patients deciding to forgo or stop active treatment for cancer. *European Journal of Palliative Care* 2005; 12(3):108. | Australia and Israel | Palliative anticancer treatment/ at EOL | All | Patients, family members, oncologists, nurses, social workers, allied health staff  (N=45) | Focus group and qualitative interviews | 1) Decisions are not a single event, they are a process  2) Decisions are taken alone  3) Decision to stop happens when treatment interferes with quality of life | LMM - **M** |
| **22** | Koedoot CG, De RJ, Stiggelbout AM, Stalmeier PF, De A, Bakker PJ et al. Palliative chemotherapy or best supportive care? A prospective study explaining patients' treatment preference and choice. *Br J Cancer* 2003; 89(12):2219-2226. | Netherlands | Palliative chemotherapy/ Chemotherapy at EOL | All | Patients (N=140) | Qualitative interview | 1) Younger patients’ preference for continuing palliative chemo. Other demographics not related  3) Expectation oncologist will propose palliative chemotherapy  4) Patient’s pre-consultation preference and actual choice are related  5) Preference for continuing:  High level internal control stronger deferring decision style, striving for length of life, low preference for participating in the decision-making | MML - **M** |
| **23** | Penson, RTF et al. ‘Attitudes to chemotherapy in patients with ovarian cancer’, *Gynecologic Oncology,* 94 (2004) 427–435. | USA and UK | Palliative chemotherapy/ Chemotherapy at EOL | Ovarian | Patients (n=122) Staff (n=37) | Questionnaire | 1) Continuation of chemotherapy on occurrence of ovarian cancer with no proven benefit  2) Patients more likely to think there is benefit in chemotherapy for recurrent ovarian cancer  3) US patients less likely to want palliative care and more likely to want chemo – i.e. patient driven | MML - **M** |
| **24** | Hirose T, Horichi N, Ohmori T, Kusumoto S, Sugiyama T, Shirai T, Ozawa T, Ohnishi T, Adachi M. Patients preferences in chemotherapy for advanced non-small-cell lung cancer. *Intern Med*. 2005 Feb;44(2):107-13. | Japan | Palliative chemotherapy/ Chemotherapy at EOL | Non-small-cell Lung Cancer | Lung cancer patients (N=73)  Control group (N=120) | Questionnaire | 1) Cancer patients would choose for 3 months of life benefit  2) Cancer patients more likely than others with similar prognosis to want to continue treatments for little benefit | MML - **M** |
| **25** | Brearley S, Craven O, Saunders M. Clinical features of oral chemotherapy: results of a longitudinal prospective study of breast and colorectal cancer patients receiving capecitabine in the UK. *European Journal of Cancer Care* 2010; 19(4):425. | UK | Capecitabine | Colorectal and breast | Patients  (N=81) | Toxicity assessments during capecitabine treatment | 1) Most common reason for discontinuation was being unfit for treatment (9.8%), which, when included alongside toxicity-related lack of fitness, resulted in over 17% of subjects discontinuing treatment | HLL - **L** |
| **26** | Sarenmalm EK, Thorén-Jönsson A, Gaston-Johansson F, Öhlén J. Making sense of living under the shadow of death: Adjusting to a recurrent breast cancer illness. *Qualitative Health Research*. 2009; 19:1116–1130. | Sweden | Palliative anticancer treatment/ at EOL | Breast | Patients (N=20) | Qualitative interview | 1) Importance of ‘hope’ Patients hoped that treatment would help or for alternative treatments. Never wanted to be told that there was nothing more to be done  2) Participants described hopes of different kinds, the most frequent hope was to survive, or if not, just to have some more time to live  3) Accepting loss and dealing with loss part of the decision | HLL - **L** |
| **27** | Voogt E, van der Heide A, Rietjens JA, van Leeuwen AF, Visser AP, van der Rijt CC, van der Maas PJ.  Attitudes of patients with incurable cancer toward medical treatment in the last phase of life. *J Clin Oncol.* 2005 Mar 20;23(9):2012-9. | Netherlands | Palliative anticancer treatment/ at EOL | All | Patients (n=122) | Questionnaire | 1) Short period of cancer more likely to want treatment  2) Younger patients were more inclined to prefer life prolongation | HLL - **L** |
| **28** | Bakitas. Proxy Perspectives Regarding End-of-life Care for Persons with Cancer. *American Cancer Society* 2008; 112:1854-1861. | USA | Palliative chemotherapy/ Chemotherapy at EOL | All | Bereaved relatives of those who died from advanced cancer (N=125) | Structured telephone survey | 1) 17% of respondents believed there relatives wishes in the last week of life were to have a course of life extending treatment  2) 78% felt their relatives wishes were followed  3) 83% felt physicians told them about treatment options in an understandable way | MLL - **L** |
| **29** | Colla CH, Morden NE, Skinner JS, Hoverman JR, Meara E. Impact of Payment Reform on Chemotherapy at the End of Life. *American Journal of Managed Care* 2012; 18(5):E200-E206. | USA | Palliative chemotherapy/ Chemotherapy at EOL | All | Medicare patients  (N=235,821) | Patient records study | 1) Chemotherapy receipt near the end of life was significantly more likely for those treated in physician office settings versus hospital out-patient departments  2) Payment reform of Medicare caused chemotherapy at the end of life for those treated in the doctor’s office to drop | MLL - **L** |
| **30** | Emanuel EJ, Young-Xu Y, Levinsky NG, Gazelle G, Saynina O, Ash AS. Chemotherapy use among medicare beneficiaries at the end of life. *Ann Intern Med* 2003;138:639– 43. | USA | Palliative chemotherapy/ Chemotherapy at EOL | All | Medicare patients | Patient records study | 1) The cancer’s responsiveness to chemotherapy does not seem to influence  whether dying patients receive chemotherapy at the end of life  2) chemo at end of life decreases with age | MLL - **L** |
| **31** | Gauthier DM, Swigart VA. The contextual nature of decision making near the end of life: hospice patients' perspectives. *American Journal of Hospice & Palliative Medicine* 2003; 20(2):121-128. | USA | Palliative anticancer treatment/ at EOL | All | Patients (n= 14) | Qualitative interview | 1) Decision making for the terminally  ill adults in this study was filtered through personal understanding, values and beliefs, life context, and relationships  2) Participants in the study adjusted and responded on a day-by-day basis.  3) Influence of physical  symptoms, pain, and decreasing physical  functioning on key aspects of the  decision-making process – made patients realise “terminality” and increased physical dependence influenced when and how decisions were made | MLL - **L** |
| **32** | Harrington SE, Smith TJ. The role of chemotherapy at the end of life: 'when is enough, enough?'. *Journal - American Medical Association* 2008; 299(22):2667-2678. | USA | -Gemcitabine  -Carboplatin  -Pemetrexed  -Intrathecal methotrexate  - Liposomal cytarabine | Lung | Lung cancer patient  (N=1) | Case study | 1) Ongoing process  2) Involves sophisticated oncological assessment, a focus on the patient’s goals of care, and a balancing of perspectives of the patient and treating oncologist  3) The oncologist had brought up  hospice, and the patient initially declined it, only accepting palliative care involvement when  death was imminent  4) Patient only felt like he was about to die when he had 2 weeks left with pneumonia | LLM - **L** |
| **33** | Hashimoto K, Yonemori K, Katsumata N, Hotchi M, Kouno T, Shimizu C et al. Factors that affect the duration of the interval between the completion of palliative chemotherapy and death. *Oncologist* 2009; 14(7):752-759. | Japan | Palliative chemotherapy/ Chemotherapy at EOL | All | Patients (N=255) | Retrospective case review | 1) Young patients who were symptomatic tended to choose chemotherapy instead of entering a palliative care unit until the very near-the-end-of-life stage | MLL - **L** |
| **34** | Kao S, Shafiq J,Vardy J, Adams: Use of chemotherapy at end of life in oncology patients*. Ann Oncol,* 20: 1555-1559, 2009. | Australia | Palliative chemotherapy/ Chemotherapy at EOL | All | Patients (N=747) | Retrospective case review | 1) Factors associated with commencement: younger age, female gender, cancer type (CNS tumours) and the chemosensitivity of the tumour  2) The only significant predictor found for continuation of palliative chemotherapy in the last 4 weeks of life was the individual treating medical oncologist  3) No factors that predicted for continuation of palliative chemotherapy in the last 2 weeks of patient’s life | MLL - **L** |
| **35** | Keam B, Oh DY, Lee SH, Kim DW, Kim MR, Im SA et al. Aggressiveness of cancer-care near the end-of-life in Korea. *Japanese Journal of Clinical Oncology* 2008; 38(5):381-386. | Korea | Palliative chemotherapy/ Chemotherapy at EOL | All | Patients (N=298) | Retrospective case review | 1) 31.2% discontinued 2 months before death, 19.1% discontinued 3 months before death and 19.1% 1 month before death  2) Agreement rate of written DNR issue and hospice referral and proportion of hospital death were not associated with the timing of discontinuation chemotherapy | MLL- **L** |
| **36** | Martoni AA, Tanneberger S, Mutri V. Cancer chemotherapy near the end of life: the time has come to set guidelines for its appropriate use. *Tumori.* 2007; 93(5):417–422. | Italy | Old-generation drugs* 56 (55.5)  New-generation drugs 37 (36.6)  Gemcitabine 20 (19.8)  Oxaliplatin 5 (5)  Capecitabine 5 (5)  Taxanes 3 (3)  Oral vinorelbine 2 (2)  Irinotecan 1 (0.9)  CT+monoclonal antibody | All | Patients (N=793) | Retrospective case review | 1) Not related to chemo-sensitivity: Use of CT in the last month of life in our study did not appear to be influenced by the tumour’s chemosensitivity: most of the patients had tumours with intermediate or low chemosensitivity | LLM - **L** |
| **37** | Morita et al. ‘Communication about the ending of anticancer treatment and transition to palliative care’, *Ann Oncol.* 2004 Oct;15(10):1551-7. | Japan | Palliative anticancer treatment/ at EOL | All | Bereaved family (N=318) | Questionnaire | 1) Physician – patient communication in private  2) Family distress moderately correlated with needing to improve | MLL - **L** |
| **38** | Zhang Y, Zyzanski J, Siminoff A. Ethnic differences in the caregiver's attitudes and preferences about the treatment and care of advanced lung cancer patients. *Psycho-Oncology* 2012; 21(11):1250. | USA | Palliative anticancer treatment/ at EOL | Lung | African American (n=26) and White (n=173) caregivers of lung cancer patients | Semi-structured questionnaire interview | 1) Denial dying: African American caregivers continued to believe that treatment was curative, and tended to be more avoidant around issues of death  2) Talking to children as support or to meet expectations  3) Children’s responsibility: African American caregivers were also less likely to agree that children have a responsibility to make treatment decisions | MLL - **L** |
| **39** | Coulehan J. "They wouldn't pay attention": Death without dignity. *American Journal of Hospice & Palliative Medicine* 2005; 22(5):339-343. | USA | Palliative chemotherapy/ Chemotherapy at EOL | Pancreatic | Pancreatic cancer patient  (N=1) | Case Study | Patient and daughter wanted to stop treatment and have palliative care. Physician wanted to continue and patient complied | LLL - **L** |
| **40** | Hui D, Con A, Christie G. Goals of care and end-of-life decision making for hospitalized patients at a Canadian tertiary care cancer center. *Journal of Pain and Symptom Management* 2009; 38(6):871. | Canada | Palliative anticancer treatment/ at EOL | Gastrointestinal 36 (30.5%)  Lung 21 (17.8%)  Breast 14 (11.9%)  Hematologic 11 (9.3%)  Gynecological 10 (8.5%)  Genitourinary 9 (7.6%)  Primary unknown 8 (6.8%)  Head and neck 7 (5.9%)  Others 2 (1.7%) | Patients (N=118) | Retrospective case review | 1) Early implemented supportive care plans, appropriateness of investigations and diagnosis of dying were associated with discontinuing treatments | LLL - **L** |
| **41** | Liu TW, Chang WC, Wang HM, Chen JS, Koong SL, Hsiao SC et al. Use of chemotherapy at the end of life among Taiwanese cancer decedents, 2001-2006. *Acta Oncologica* 2012; 51(4):505-511. | Taiwan | Palliative chemotherapy/ Chemotherapy at EOL | All | Patients  (n=204850) | Retrospective case review | Factors associated with continuing chemo at EOL:  1) Gender: Male more likely to receive chemotherapy  2) Age: Continuation of chemotherapy in the last month of life decreased sharply with age and had age gradients  3) Marital status  4) Comorbidity level progressively decreased the odds of using chemotherapy in the last month of life  5) Primary site: Compared to patients with lung cancer (Taiwan ’ s leading cause of cancer death), patients with haematological malignancies and breast cancer were significantly more likely to receive chemotherapy  6) Length of time after diagnoses  7) Cancer patients cared for by a medical oncologist as their primary physician  8) Hospital factors | LLL - **L** |
| **42** | Yun YH et al. ‘Chemotherapy use and associated factors among cancer patients near the end of life’, *Oncology*. 2007;72(3-4):164-71. | Korea | Palliative chemotherapy/ Chemotherapy at EOL | All | Patients | Retrospective case review | Factors associated with chemo at the end of life:  1) The frequency of chemotherapy use was lower for older patients. In those ≧65 years old, there was no difference between women and men in the proportion that received chemotherapy  2) For patients <65 years of age, a larger proportion of women than men received chemotherapy, and chemotherapy use was significantly less frequent for patients with refractory disease than for those with responsive disease  3) Patients dying at a relatively small hospital without a hospice inpatient unit were significantly more likely to receive chemotherapy | LLL – **L** |
